# Correction: Zr–Cu alloy filler metal for brazing SiC ceramic

**DOI:** 10.1039/c8ra90065e

**Published:** 2018-07-31

**Authors:** Bofang Zhou, Keqin Feng

**Affiliations:** School of Manufacturing Science and Engineering, Sichuan University Chengdu Sichuan 610065 P. R. China hs_zbf@163.com; School of Materials Science and Engineering, Hubei University of Automotive Technology Shiyan Hubei 442002 P. R. China; Sichuan Engineering Technical College Deyang Sichuan 618000 P. R. China

## Abstract

Correction for ‘Zr–Cu alloy filler metal for brazing SiC ceramic’ by Bofang Zhou *et al.*, *RSC Adv.*, 2018, **8**, 26251–26254.

In the original publication, errors were present in affiliation *b*, the corrected version is shown below.

The Royal Society of Chemistry apologises for these errors and any consequent inconvenience to authors and readers.

## Supplementary Material

